# Enantioselective Separation and Negative Transport of Chiral Particles via Optical Needle Fields

**DOI:** 10.1002/nap2.70189

**Published:** 2026-06-27

**Authors:** Manman Li, Xu Chen, Shaohui Yan, Qiang Zhang, Wenyu Gao, Yuan Zhou, Xiaohao Xu, Baoli Yao

**Affiliations:** ^1^ State Key Laboratory of Ultrafast Optical Science and Technology Xi'an Institute of Optics and Precision Mechanics Chinese Academy of Sciences Xi'an China; ^2^ University of Chinese Academy of Sciences Beijing China

**Keywords:** chiral sorting, chirality, optical forces, optical needle, optical pulling

## Abstract

Chirality is a fundamental property of life and a critical determinant of material properties, making the development of efficient, rapid, and nondestructive chiral detection and separation methods imperative. Here, we advance beyond conventional two‐dimensional chiral manipulation by introducing a tailored optical needle field that enables enantioselective, long‐range, and three‐dimensional control. Generated by the tight focusing of an annular beam, this unique field retains chiral sensitivity and facilitates the simultaneous enantioselective separation, size‐based sorting, and optical pulling of chiral particles. Moreover, we engineer a dual‐needle configuration that achieves the spatially separated and concurrent negative transport of both enantiomers within a unified system, offering tunable separation and transport distances. By integrating optical pulling with chiral light–matter interactions, this work establishes a robust and versatile platform for all‐optical, high‐throughput chiral sorting, with promising applications in pharmaceutical science, biochemical sensing, and nanotechnology.

## Graphical abstract

This work proposes an all‐optical strategy that leverages a tailored optical needle field for the enantioselective separation and 3D manipulation of chiral particles. By introducing phase modulation, we engineer a dual‐needle configuration capable of spatially separating and negatively transporting both enantiomers within a single system, offering a promising approach for high‐throughput chiral sorting.

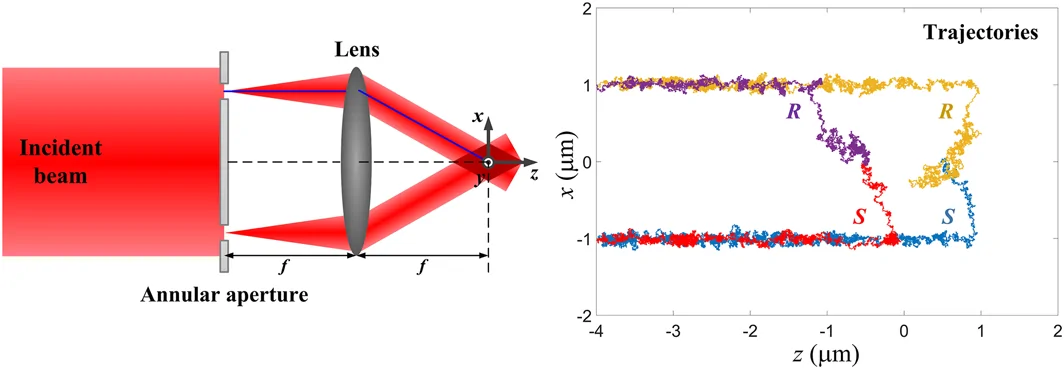

## 1 Introduction

Chirality, the geometric property of an object that cannot be superimposed onto its mirror image by any rotations or translations, underlies a vast array of natural phenomena across scales from subatomic to galactic levels [1, 2, 3]. Although formally defined over a century ago, this concept continues to stimulate extensive research due to its fundamental importance across physics, chemistry, and biology. This geometric distinction gives rise to enantiomers—molecular mirror twins that share identical chemical formulas yet differ in spatial arrangement. Such subtle divergence can lead to dramatically different chemical properties and biological functions [4]. A tragic example is the thalidomide disaster, where one enantiomer offered therapeutic benefit, whereas its mirror counterpart caused severe teratogenic effects [5]. Furthermore, the majority of modern pharmaceuticals are chiral, and their synthesis typically results in a racemic mixture of both enantiomers, subsequently necessitating separation [6]. Current methods for chiral detection, such as polarimetry, chiral Raman spectroscopy, and high‐performance liquid chromatography, are well established [7, 8, 9]. However, limitations including sensitivity to interfering factors, potential sample damage, and operational complexity can compromise their accuracy and practicality. Consequently, the development of efficient, rapid, accurate, and nondestructive techniques for the detection and separation of chiral molecules remains a central pursuit in the scientific research, with profound implications for pharmaceutical science, chemical synthesis, and biomedical engineering.

Recent advances in chiral light–matter interaction have opened promising avenues to address this challenge [10, 11]. When light carries momentum that is transferred to microscopic particles, it generates optical forces capable of driving controlled motion, known as optical manipulation [12]. Introducing chirality into this interaction unlocks a pivotal mechanism: the emergence of distinct optical forces acting on opposite enantiomers [13]. These chirality‐dependent optical forces have garnered significant attention due to their potential for enabling enantioselective separation and chiral identification at the single‐particle level [14, 15, 16, 17, 18, 19, 20, 21, 22, 23, 24, 25, 26, 27, 28, 29, 30, 31, 32]. For instance, they enable the opposite deflection of paired enantiomers [14, 15, 16, 17, 18], the selective trapping of one enantiomer while repelling its counterpart [19, 20, 21, 22, 23], and the handedness‐dependent directional rotation of particles [24, 25, 26, 27]. Nevertheless, most existing strategies are inherently confined to two‐dimensional manipulation perpendicular to the light propagation direction [14, 15, 16, 17, 18, 19, 20, 21, 22, 23, 24, 25, 26] and often rely on additional microfluidics environments or microstructures to realize particle transport [28, 29, 30, 31, 32]. Optical pulling—a counterintuitive phenomenon in which particles are drawn backward against the direction of light propagation—stems from the intriguing physics of light‐momentum transfer and has broadened the scope for multidirectional optical manipulation [33]. However, despite the growing interest in extending this effect to chiral particles [34, 35, 36, 37, 38], it remains challenging to achieve long‐range, simultaneous transport of both enantiomers in a single system without relying on external conditions.

In this work, we propose an all‐optical strategy for the enantioselective separation and three‐dimensional optical manipulation of chiral particles, leveraging a tailored optical needle field. This field is generated by tightly focusing an annular beam, which creates an extended region of uniform intensity while preserving the chiral sensitivity. We demonstrate its capability for the simultaneous enantioselection and size‐based sorting of particles, as well as their optical pulling. Furthermore, by introducing phase modulation, we extend this concept to a dual‐needle field. This advanced configuration enables the spatially separated and concurrent negative transport of both enantiomers within a single system, offering a promising approach for high‐throughput chiral sorting.

## 2 Results and Discussion

### 2.1 Generation of Optical Needle Fields

The schematic diagram of the system for generating the optical needle field is depicted in Figure 1a. In this setup, an incident beam is transmitted through an annular aperture and subsequently focused by a high numerical aperture (NA) objective lens. The annular aperture serves as an amplitude modulator, removing small‐angle components and preserving large‐angle components of the incident beam, and thereby facilitates a significant extension of the depth of focus (DOF). Within the Richards–Wolf vectorial diffraction theory [39, 40], the optical field at the target particle is:

(1) $$E_{\text{inc}}\left(r\right)=\frac{-ik_{1}f}{2\pi }\int_{\theta_{\min}}^{\theta_{\max}}\int_{0}^{2\pi }\sqrt{\cos\;\theta }L\left(\theta ,\phi \right)\exp\left(ik_{1}\cdot r\right)\sin\;\theta d\phi d\theta ,$$

where *k*_1_ = 2π*n*_1_/*λ* is the image‐space wave number, with *n*_1_ denoting the corresponding refractive index and *λ* the free‐space wavelength; *f* is the focal length; and **k**_1_ and **r** are the image–space wave vector and observation point coordinate, respectively. The input field follows **L**(*θ*, *ϕ*) = **u** *l*(*θ*), with polarization vector **u** and with radial envelope *l*(*θ*) given by

(2) $$l\left(\theta \right)=l_{0}\;\exp\left[-\beta_{0}^{2}{\left(\frac{\sin\;\theta }{\sin\;\theta_{\max}}\right)}^{2}\right],\theta_{\min}\leq \theta \leq \theta_{\max}\;.$$

Here, *l*_0_ is a coefficient dependent on the input power *P*, and *β*_0_—defined as the ratio of the pupil radius to the beam waist—is set to 1.5. The convergence angles *θ*_min_ and *θ*_max_ denote the minimum and maximal angles, determined by the width of the annular aperture and the NA of the objective lens.

**FIGURE 1:**
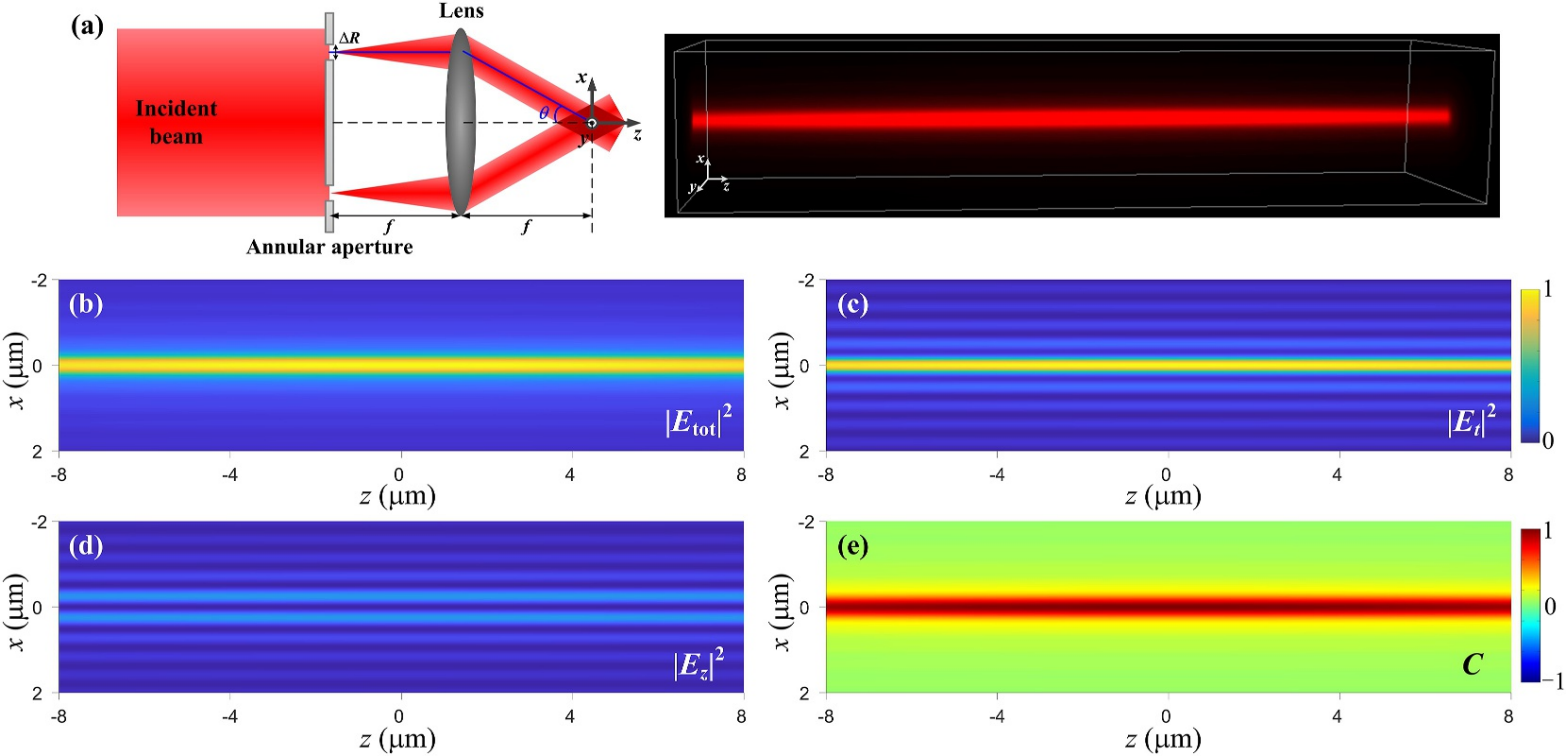
Schematic and distributions of the optical needle field. (a) Generation schematic. Normalized distributions in the *xz*‐plane of the (b) total electric intensity, (c) transverse, (d) longitudinal electric components, and (e) optical chirality density.

To reveal the optical properties of the focused left‐handed circularly polarized beam, the distributions of the total intensity, transverse component, and longitudinal component of the electric fields in the *xz*‐plane are presented in Figure 1b–d, respectively. The calculation parameters are: *p* = 100 mW, *λ* = 1.064 μm, *n*_1_ = 1.33, NA = 1.26, and *θ*_min_ = 0.99*θ*_max_. The intensity profile confirms the successful generation of the optical needle field. This characteristic is primarily attributed to the transverse component governed by the Bessel function *J*_0_(*ρ*), which peaks at *ρ* = 0, as can be seen from the analytical expression for the focused field provided in Supporting Information S1: Equation S1. The calculated transverse and longitudinal full widths at half maximum (FWHM) of the field are 0.44*λ* and 51.22*λ*, respectively. Moreover, as illustrated in Supporting Information S1: Figure S1, the DOF increases with *θ*_min_, since a larger *θ*_min_ causes the focused field to more closely approximate a nondiffracting Bessel beam. With *θ*_min_ = 0.99*θ*_max_ and a 2 mm focal length, the computed annulus aperture width of Δ*R* ≈ 8.2 μm is well above the operational wavelength, ensuring the design's practicality. Furthermore, the magnetic field, derived from Maxwell's equation ∇ × **E** = *iωμ* **H**, exhibits a normalized intensity distribution identical to that of the electric field components, albeit with a π/2 phase difference between corresponding electric and magnetic field components. Based on the optical chirality density *C* ∝ Im[**E**·$H^{*}$] [41], the optical chirality present in the optical needle field is demonstrated, as also visualized by its normalized density distribution in Figure 1e. Notably, the chirality density is uniformly positive, indicating that the field retains the same handedness as the incident circularly polarized beam. Consequently, this longitudinally tolerant, chirality‐carrying optical needle field shows significant potential for applications such as chiral particle sorting.

### 2.2 Optical Forces on Chiral Particles

Within the framework of Mie theory, the interaction between the engineered optical needle field and a chiral particle is modeled. The particle scatters the incident fields (**E**_inc_, **H**_inc_) to produce external scattered fields (**E**_sca_, **H**_sca_), experiencing a time‐averaged optical force 〈F〉. This force is calculated via the surface integral of the Maxwell stress tensor **T̅**,

(3) $$\langle F\rangle =\langle \oint_{S}\hat{n}\cdot \bar{T}ds\rangle \;.$$

Here, $\hat{n}$ is the outward normal to the closed surface *S* enclosing the particle. The tensor is defined as **T̅** = *ε*_1_ *ε*_0_ **EE** + *μ*_1_ *μ*_0_ **HH** − 1/2(*ε*_1_ *ε*_0_ *E* ^2^ + *μ*_1_ *μ*_0_ *H* ^2^)**I̅**, with *ε*_0_, *μ*_0_ the vacuum permittivity and permeability, *ε*_1_, *μ*_1_ the relative permittivity and permeability of the ambient medium, **I̅** the unit dyadic, and **E** (= **E**_inc_ + **E**_sca_) and **H** (= **H**_inc_ + **H**_sca_) the total external fields. The scattering is solved using Waterman's T‐matrix method [42], yielding the optical force (see derivation in Supporting Information S1: Section 2) expressed in terms of field expansion coefficients.

We consider a chiral particle characterized by a relative permittivity of *ε*_2_ = 2.53, a permeability of *μ*_2_ = 1, and a chirality parameter *κ*. Note that the *κ* used herein is an effective geometric chirality parameter for artificially structured microparticles, typically ranging from −1 to 1 [14, 16, 17, 28], which differs distinctly from the intrinsic chirality parameter of natural molecules (typically on the order of 10^−5^). The negative and positive values of *κ* correspond to the R (right‐handed) and S (left‐handed) enantiomers, respectively. Figure 2a–c display the distributions of the optical forces in the *xz*‐plane for the S (*κ* = 0.5) and R (*κ* = −0.5) enantiomers, as well as an achiral particle (*κ* = 0) with a radius of *a* = 0.25 μm, generated by the optical needle field. The arrows represent the optical force vector, whereas the background color indicated the magnitude of the optical force, defined as ${\left(F_{x}^{2}+F_{z}^{2}\right)}^{1/2}$. Notably, the S enantiomer and the achiral particle can be transversely trapped on the optical axis, whereas the R enantiomer is repelled, demonstrating a handedness‐selective trapping mechanism. In addition to the transverse component, the particles also experience a longitudinal optical force directed along the negative optical axis, manifesting as an optical pulling force. The variations of the transverse and longitudinal optical forces along the *x*‐, *y*‐ and *z*‐axis are plotted in Figure 2d,e, respectively. The transverse optical force profiles along the *x* and *y* directions coincide due to the rotational symmetry of the incident optical field and the spherical particle. Relative to the achiral particle, the S enantiomer experiences stronger optical forces because a chirality‐dependent component, arising from its matched handedness with the field, adds constructively to the baseline force. Conversely, the R enantiomer experiences weaker forces because the chirality‐dependent force has the opposite sign.

**FIGURE 2:**
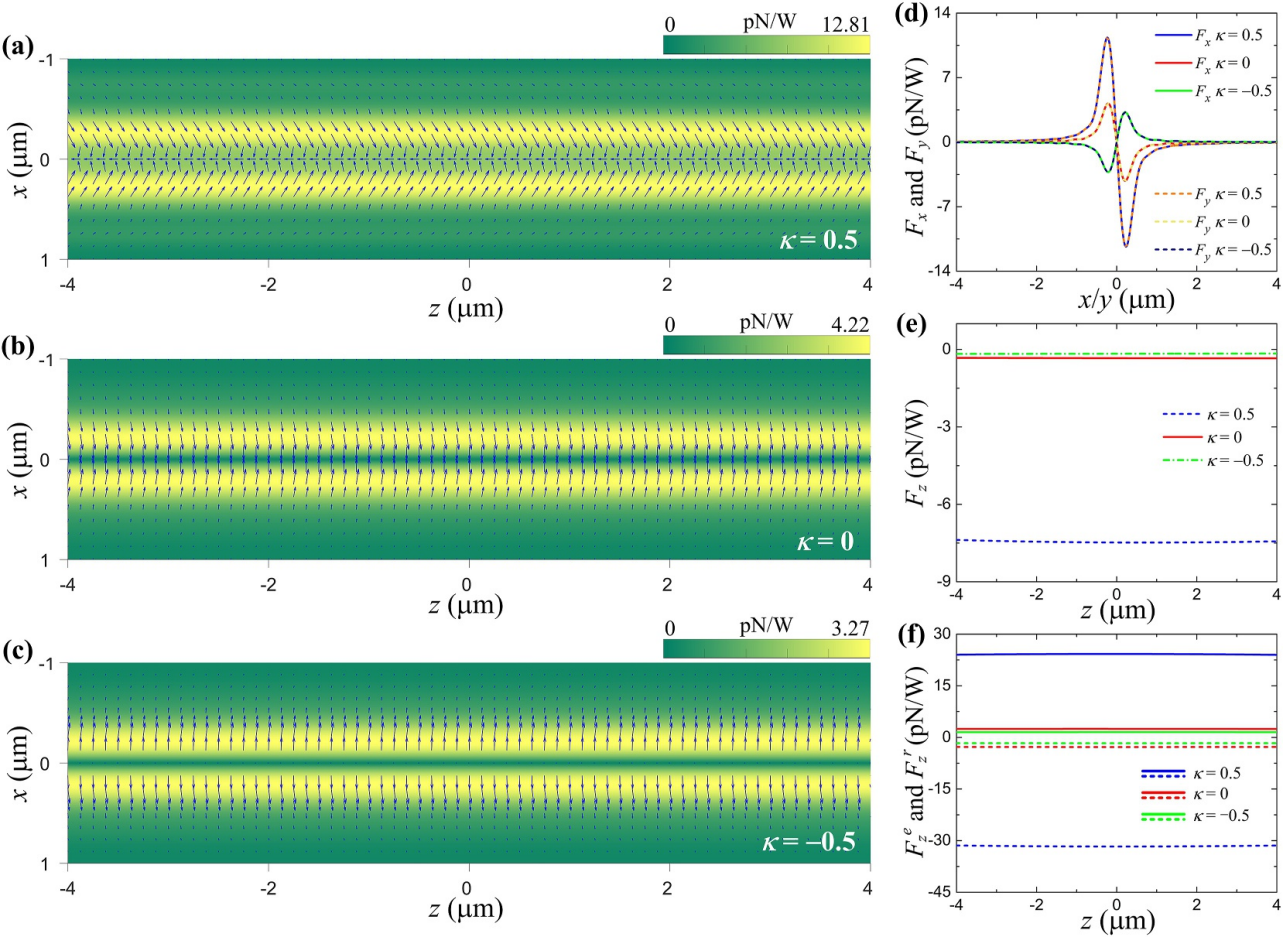
Mapping of optical force: (a–c) Distributions in the *xz*‐plane for the S enantiomer, achiral particle, and R enantiomer. (d–f) Corresponding positional dependencies of the (d) transverse force, (e) longitudinal force, and (f) longitudinal extinction (solid) and recoil (dashed) forces.

To further elucidate the mechanism of the optical pulling force, the longitudinal optical force in Supporting Information S1: Equation S8 can be decomposed into two distinct parts: the extinction force $<F_{z}^{e}>$ and the recoil force $<F_{z}^{r}>$. The extinction force arises from the interaction between the incident and scattered fields, whereas the recoil force stems from the momentum transfer during scattering. These two components are given by:

(4) $$\begin{array}{l} <F_{z}^{e}>=-\frac{\varepsilon_{1}}{2k_{1}^{2}}\sum_{n=1}^{\infty }\sum_{m=-n}^{n}\text{Im}\left[c_{4}\left(a_{m,n+1}p_{m,n}^{*}+p_{m,n+1}a_{m,n}^{*}+b_{m,n+1}q_{m,n}^{*}+q_{m,n+1}b_{m,n}^{*}\right)+ic_{5}\left(q_{m,n}a_{m,n}^{*}+b_{m,n}p_{m,n}^{*}\right)\right],\\ <F_{z}^{r}>=-\frac{\varepsilon_{1}}{k_{1}^{2}}\sum_{n=1}^{\infty }\sum_{m=-n}^{n}\text{Im}\left[c_{4}\left(p_{m,n+1}p_{m,n}^{*}+q_{m,n+1}q_{m,n}^{*}\right)+ic_{5}q_{m,n}p_{m,n}^{*}\right], \end{array}$$

where (*a*_*mn*_, *b*_*mn*_) and (*p*_*mn*_, *q*_*mn*_) are the expansion coefficients of the incident and scattered fields, *c*_4_ = {*n*(*n* + 2) (*n* − *m* + 1) (*n* + *m* + 1)/[(*n* + 1)^2^(2*n* + 1) (2*n* + 3)]}^1/2^, *c*_5_ = *m*/[*n*(*n* + 1)]. Given that the extinction force is intrinsically positive, a net optical pulling force necessitates a recoil force that is both negative and greater in magnitude. This condition is satisfied, as illustrated in Figure 2f, which displays the longitudinal extinction and recoil forces for the three particle types. Moreover, the matched chirality of the S enantiomer further enhances forward momentum scattering, leading to a stronger negative recoil force and thus a more pronounced pulling effect than the others. Consequently, this optical needle field enables enantioselective transport by simultaneously trapping and pulling the matched S enantiomer backward. Notably, the R enantiomer can be similarly transported by switching the incident field to right‐handed circular polarization, allowing selective transport of either enantiomer as needed. It is worth noting that at *λ* = 1.064 μm, typical dielectric microparticles exhibit weak dispersion and negligible absorption. Dispersion merely modulates the optical force magnitude without altering the sign of enantioselective forces, whereas weak absorption may induce particle rotation but cannot alter the center‐of‐mass sorting result. Thus, the main conclusions remain robust under realistic material conditions.

Figure 3 systematically investigates the influence of the chirality parameter and particle radius on the longitudinal optical force. In Figure 3a, the colored and white regions map parameter combinations that yield optical pulling and pushing forces, respectively. The results reveal that a net pulling force arises only within a specific window where size and chirality are mutually constrained. Moreover, the force's dependence on radius exhibits two distinct regimes governed by chirality (Figure 3b): for *κ* ≤ 0.345, the force switches from push to pull and back to push; for *κ* > 0.345, the pulling force increases, then decreases, and eventually reverts to pushing. Similarly, the chirality dependence is strongly size‐contingent (Figure 3c): with small particles (*a* ≤ 0.225 μm), the force changes from weak pushing to strengthening pull; with mid‐sized particles (0.225–0.27 μm), a stable pulling force intensifies with chirality; and with larger particles (0.27–0.34 μm), the pulling force first strengthens, then weakens, and finally switches to pushing. This results demonstrate a viable strategy for the simultaneous enantioselection and size‐based sorting of chiral particles.

**FIGURE 3:**
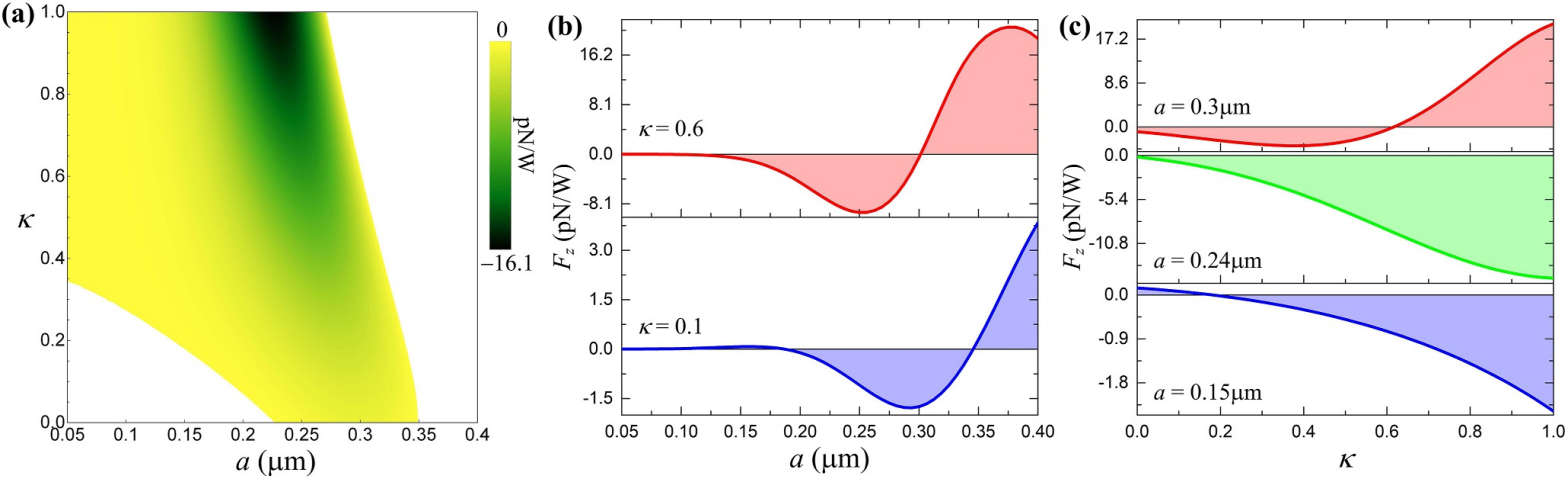
Examination of the longitudinal optical force dependence on particle properties. (a) Force mapped against chirality parameter and particle radius. (b) Force profiles as a function of radius for various chirality parameters. (c) Force profiles as a function of chirality parameter for different radii.

To manipulate both enantiomers simultaneously, a dual optical needle field with opposite chirality is engineered via input‐field modulation. Leveraging the Fourier transform property of the Richards–Wolf integral in Equation (1), focal spots can be spatially shifted through pupil‐plane phase modulation [43, 44]. Accordingly, an input field that generates two *z*‐propagating optical needles is designed as:

(5) $$L\left(\theta ,\phi \right)=\frac{1}{\sqrt{2}}\left[u_{L}e^{-ik_{1}\left(x_{1}\;\sin\;\theta \;\cos\;\phi +y_{1}\;\sin\;\theta \;\sin\;\phi \right)}+u_{R}e^{-ik_{1}\left(x_{2}\;\sin\;\theta \;\cos\;\phi +y_{2}\;\sin\;\theta \;\sin\;\phi \right)}\right]l\left(\theta \right),$$

where **u**_**L**_ and **u**_**R**_ denote the left‐ and right‐handed circular polarization vectors, and (*x*_*i*_, *y*_*i*_) are the lateral displacements in the pupil plane. In this work, we consider both optical needles confined to the *xz*‐plane (i.e. *y*_1_ = *y*_2_ = 0), with their positions set to (*x*_1_, *x*_2_) = (−1, 1) μm, as shown in Figure 4. Figure 4a,b confirm that the resulting focused field constitutes a double optical needle structure, exhibiting two distinct chirality densities: a positive (left‐handed) chirality density at *x* = −1 μm and a negative (right‐handed) chirality density at *x* = 1 μm. Although interference breaks the symmetry of the focused electric field, the chirality density distribution remains symmetric. This originates from the fact that the π/2 phase difference and orthogonal vector relationship of circularly polarized beams are preserved for every constituent angular spectrum component. Interference modulates **E** and **H** synchronously, yet Im(**E**·$H^{*}$) retains a symmetric spatial distribution. The corresponding optical force distributions acting on the S and R enantiomers (*a* = 0.25 μm, *κ* = ± 0.5) in the *xz*‐plane are shown in Figure 4c,d, respectively. As anticipated, the two enantiomers are effectively separated into distinct spatial regions and pulled backward: the S enantiomer is trapped on the left‐handed optical needle at *x* = −1 μm, and the R enantiomer on the right‐handed one at *x* = 1 μm. Moreover, the optical forces acting on both enantiomers are identical in magnitude. The robustness of the selective trapping and pulling mechanism against changes in the separation distance between the two optical needles is evaluated in Supporting Information S1: Figure S2. These results confirm the enantioselective transport mechanism and demonstrate flexible separation control by tuning the interneedle distance. Given these simulation results, all key optical components required for the proposed configuration are commercially mature and routinely used in holographic optical tweezers. Although practical implementation requires precise optical alignment and system optimization, no fundamental physical barriers exist to experimentally validating this enantioselective sorting mechanism.

**FIGURE 4:**
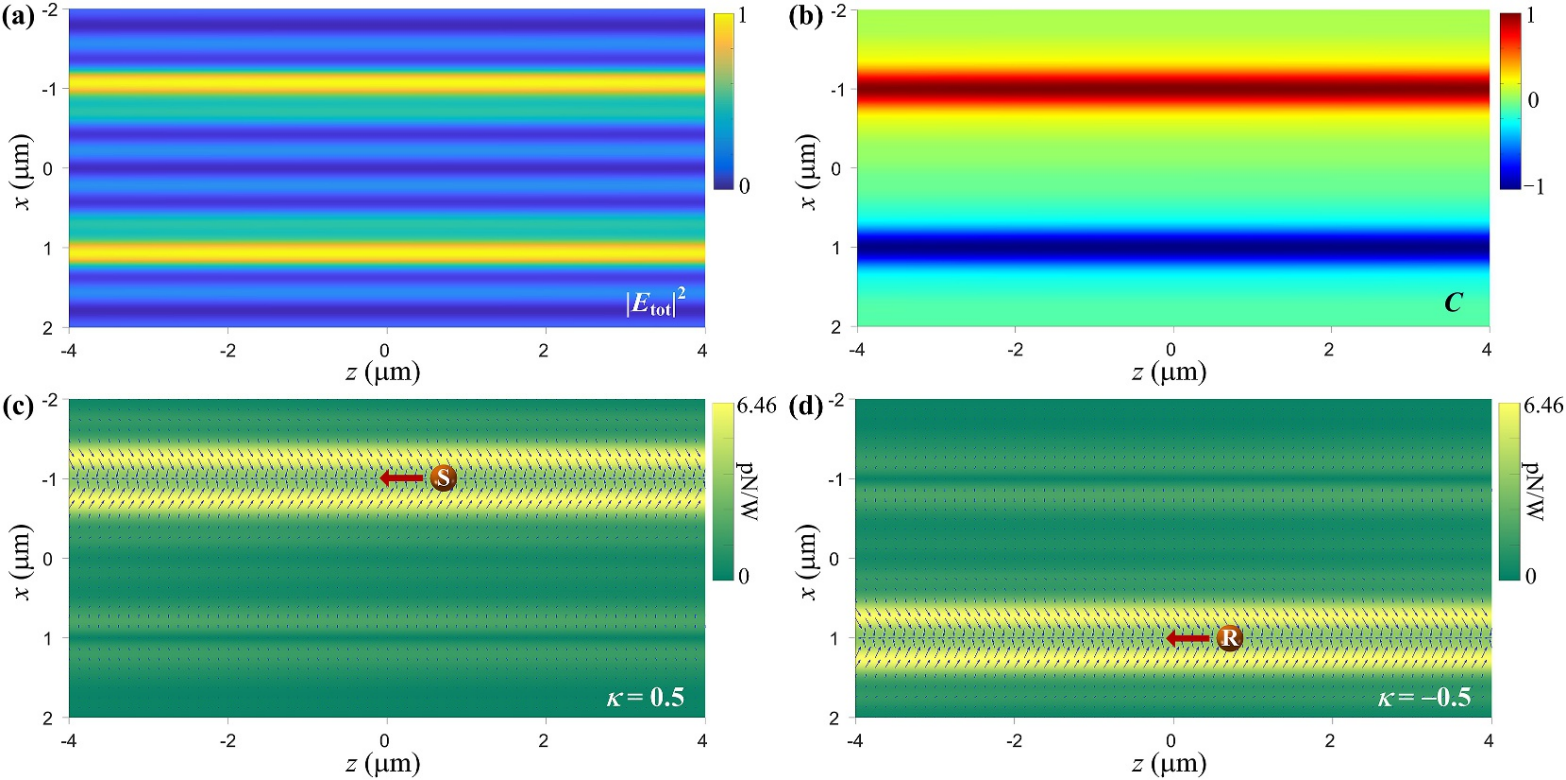
Simultaneous optical manipulation of both enantiomers. Normalized electric intensity (a) and optical chirality density (b) of the dual optical needle field in the *xz*‐plane. Corresponding optical force distributions for the S (c) and R (d) enantiomers.

### 2.3 Optically Driven Motion of Chiral Particles

In a realistic fluidic environment, the motion of chiral particles is governed by optical forces, viscous drag, and Brownian fluctuations. Their trajectories are therefore described by the overdamped Langevin equation [45, 46]:

(6) $$\frac{dr}{dt}=\frac{1}{\gamma }F\left(r\right)+\sqrt{2D}\xi \left(t\right),$$

where *D* = *k*_*B*_T/*γ* is the diffusion coefficient, *γ* = 6*πηa* is the Stokes drag coefficient, *η* is the dynamic viscosity, and ***ξ*** (*t*) is a zero‐mean, delta‐correlated Gaussian white‐noise. Given the spatial complexity of the optical force field **F**(**r**), we numerically solve Equation (6) using the Euler–Maruyama integration scheme to generate particle trajectories [47], as detailed in Supporting Information S1: Section 3.

Figure 5a,b present the simulated trajectories of four enantiomer pairs (*a* = 0.25 μm, *κ* = ± 0.5 and ± 0.1) starting at varied initial positions in the *xz*‐plane under the dual optical needle field. The simulation parameters are as follows: dynamic viscosity *η* = 0.89 mPa·s, temperature *T* = 298 K, and time step 0.5 μs. As predicted by the force profiles in Figure 4, all S enantiomers migrate toward *x* = −1 μm and all R enantiomers toward *x* = 1 μm, after which both types are pulled along the negative optical axis and eventually converge at *z* = −4 μm. For the higher chirality case (*κ* = ± 0.5, Figure 5a), the simulation spans a duration of 0.1 s. The transverse separation between enantiomers is controllable by adjusting the optical needle positions, as exemplified by trapping at *x* = ± 3 μm followed by negative transport in Supporting Information S1: Figure S3a. The longitudinal transport distance is also tunable, enabling long‐range transport from *z* = 26 μm to *z* = −28 μm, as shown in Supporting Information S1: Figure S3b.

**FIGURE 5:**
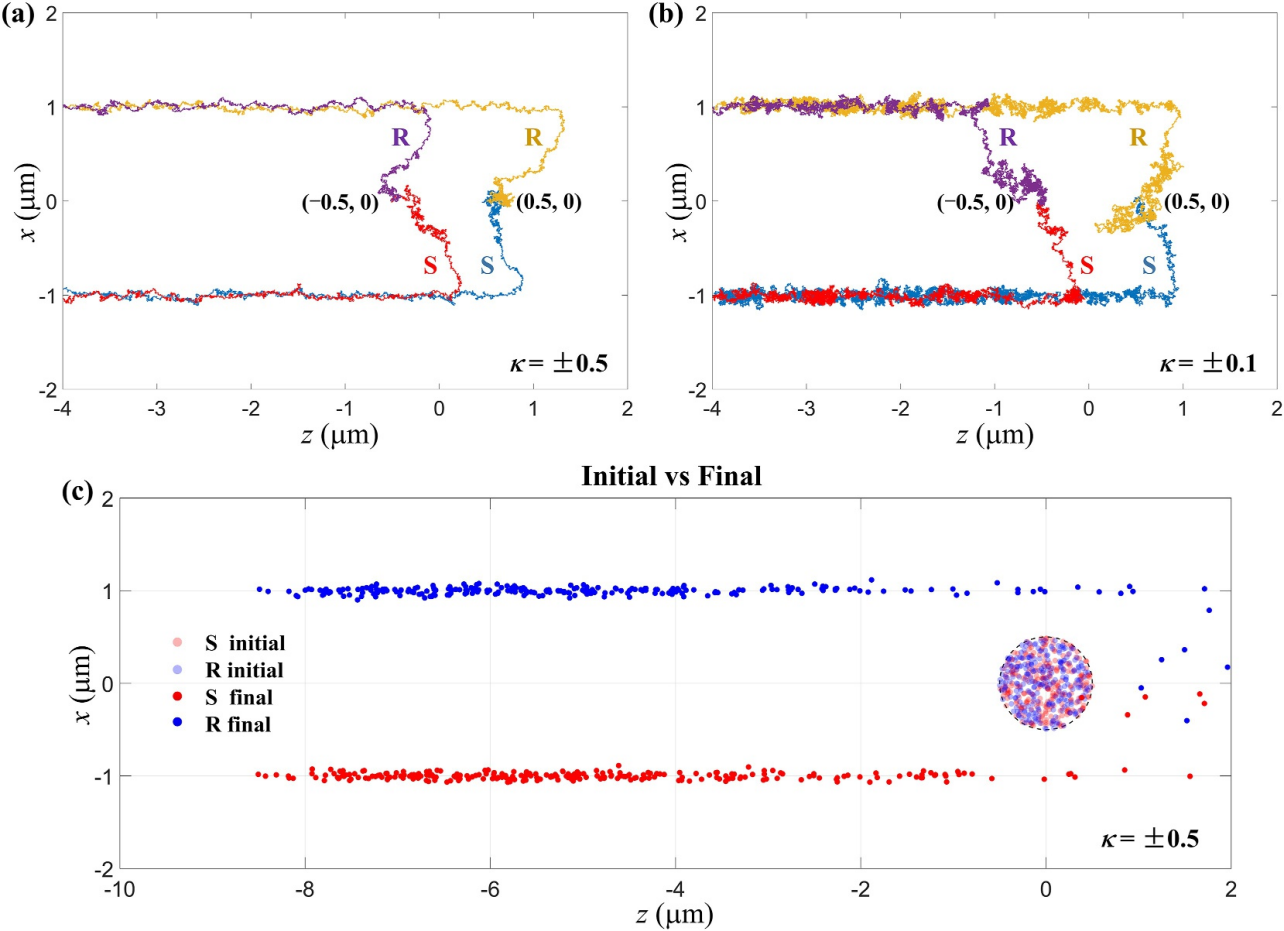
Motion simulations of enantiomer pairs in the *xz*‐plane. (a, b) Trajectories of enantiomer pairs starting from (−0.5, 0) and (0.5, 0) μm for *κ* = ± 0.5 (a) and *κ* = ± 0.1 (b), with S enantiomers (red, blue) and R enantiomers (purple, orange). (c) Ensemble simulation of 500 particles (250 S, 250 R, *κ* = ± 0.5) after 0.1 s, with initial random (semi‐transparent) and final (solid) positions.

For the lower chirality case (*κ* = ± 0.1, Figure 5b), the weaker chiral response yields a reduced optical force, necessitating a longer simulation time (0.5 s) to achieve comparable transport. Since the optical force scales linearly with input power, it can be enhanced by increasing the power accordingly. We further simulated enantiomers with even lower chirality (*κ* = ± 0.05, *a* = 0.25 μm) or smaller size (*a* = 0.15 μm, *κ* = ± 0.5), using a moderately elevated power of 200 mW (Supporting Information S1: Figure S3c,d). Effective enantioselective separation and negative transport are achieved in both instances. These results collectively confirm that the diminished optical forces arising from low chirality or small particle size can be at least partially compensated by prolonging the interaction time or raising the input power.

To obtain a statistical quantification of sorting performance, we simulated 500 randomly initialized particles (250 S + 250 R) under the same conditions as in Figure 5a. Each particle's starting position was randomized within a circular region of radius 0.5 μm. Figure 5c shows the initial (semitransparent) and final (solid) particle positions. After 0.1 s, the initially dispersed particles exhibit distinct separation: 97.6% of S enantiomers and 97.2% of R enantiomers are transversely confined to their corresponding target optical needles, whereas both populations propagate along the negative *z*‐axis concurrently. Over this duration, many enantiomers reach final *z*‐positions more negative than −4 μm, with several falling below −8 μm. These statistical results quantitatively verify the robust separation capability and promising high‐throughput potential of the proposed dual‐needle configuration. Ultimately, this demonstrates that the optical forces in our system are sufficient to overcome viscous drag and Brownian motion, thereby enabling robust chiral separation and negative transport.

## 3 Conclusion

In summary, we have demonstrated a mechanism for high‐throughput enantioselective separation and optical pulling of chiral particles using a tailored optical needle field. This field, generated by the tight focusing of an annular beam within an optimized angular range, maintains high intensity uniformity over a propagation distance exceeding 50 wavelengths while retaining its chiral sensitivity. This enables the selective trapping and negative transport of a target enantiomer. The pulling action stems from a chirality‐induced enhancement of forward momentum scattering—a process governed by both particle chirality and size—which inherently facilitates simultaneous enantioselection and size‐based sorting. Through phase modulation, we further engineered a dual optical needle field capable of simultaneously accommodating both enantiomers, thereby achieving their effective spatial separation and concurrent negative transport within a single system, with both transverse separation and longitudinal transport distances being flexibly tunable. This work uniquely integrates the concepts of optical pulling with chiral light–matter interaction, offering a promising platform for advanced chiral sorting with significant potential in drug development, biochemical sensing, and nanomaterial assembly.

## Author Contributions

**Manman Li:** conceptualization, investigation, writing – original draft, methodology, validation. **Xu Chen:** writing – original draft, investigation, conceptualization, supervision. **Shaohui Yan:** methodology, software, supervision. **Qiang Zhang:** formal analysis, software. **Wenyu Gao:** visualization, validation. **Yuan Zhou:** visualization, data curation. **Xiaohao Xu:** conceptualization, investigation. **Baoli Yao:** funding acquisition, project administration, resources, writing – review and editing.

## Conflicts of Interest

The authors declare no conflicts of interest.

## Supporting information


Supporting Information S1

## Data Availability

The data that support the findings of this study are available from the corresponding author upon reasonable request.
