# Supplementary material for: Enantioselective Separation and Negative Transport of Chiral Particles via Optical Needle Fields
Source: Nanophotonics. 2026 Jun 27;15(13):e70189. doi: 10.1002/nap2.70189 (PMC13642063; doi:10.1002/nap2.70189)
Supplement: Supplementary file 1 [file NAP2-15-e70189-s001.docx]

Supporting Information

Enantioselective Separation and Negative Transport of Chiral Particles via Optical Needle Fields

Manman Li^1^, Xu Chen^1,^*, Shaohui Yan^1^, Qiang Zhang^1,2^, Wenyu Gao^1,2^, Yuan Zhou^1^, Xiaohao Xu^1^, and Baoli Yao^1,2,^*

^1^State Key Laboratory of Ultrafast Optical Science and Technology, Xi’an Institute of Optics and Precision Mechanics, Chinese Academy of Sciences, Xi’an 710119, China

^2^University of Chinese Academy of Sciences, Beijing 100049, China

*E-mail: chenxu@opt.ac.cn, yaobl@opt.ac.cn

**This PDF file includes the following sections:**

Supplementary Section 1: The properties of the focused fields

Supplementary Section 2: Optical forces exerted on chiral particles

Supplementary Section 3: Move trajectories of chiral particles

**Supplementary Section 1:** **The p****roperties of the focused fields**

Performing the azimuthal integration in Equation 1, we arrive at the expressions for the three cylindrical field components of the incident left-handed circularly polarized **u** = (**e***_x_*+*i***e***_y_*) / 2^1/2^ beam at the point (*ρ_s_*, *φ_s_*, *z_s_*):

 (S1)

Here, *A* = *k*_1_*f*/2, *β* = *k*_1_*ρ_s_*sin*θ*, and *J_m_*(*β*) denotes the *m*th-order Bessel function of the first kind.

To systematically investigate the influence of the annular width on the focused field, Figure S1 represents the total electric field intensity distributions in the *xz*-plane for *θ*_min_ values of 0.9, 0.93, 0.95, 0.97, and 0.99*θ*_max_. The results demonstrate that increasing *θ*_min_ leads to a gradual broadening of the transverse full widths at half maximum (FWHM) and a substantial extension of the longitudinal FWHM. Quantitatively, the transverse FWHMs are measured as 0.422*λ*, 0.428*λ*, 0.433*λ*, 0.438*λ*, and 0.444*λ*, while the corresponding longitudinal FWHMs are 5.82*λ*, 8.21*λ*, 11.40*λ*, 18.74*λ*, and 51.22*λ*. This behavior arises because a larger *θ*_min_ causes the focused field to more closely approximate a non-diffracting Bessel beam.

**Figure S1.** Normalized total electric intensity distributions in the *xz*-plane as a function of *θ*_min_.

**Supplementary Section 2:** **Optical forces exerted on chiral particles**

In the T-matrix method, both the incident and scattered fields are expanded in terms of vector spherical wave functions (VSWFs) as follows:

$\begin{matrix} \mathbf{E}_{inc}(\mathbf{r})=\sum_{n=1}^{\infty} \sum_{m=-n}^{n} \left[a_{mn}\mathbf{M}_{mn}^{1}(k_{1}\mathbf{r})+b_{mn}\mathbf{N}_{mn}^{1}(k_{1}\mathbf{r})\right] , \\ \mathbf{E}_{sca}(\mathbf{r})=\sum_{n=1}^{\infty} \sum_{m=-n}^{n} \left[p_{mn}\mathbf{M}_{mn}^{3}(k_{1}\mathbf{r})+q_{mn}\mathbf{N}_{mn}^{3}(k_{1}\mathbf{r})\right] . \end{matrix}$ (S2)

Here, the coefficients (*a_mn_*, *b_mn_*) and (*p_mn_*, *q_mn_*) correspond to the expansions of the incident and scattered fields, respectively. The functions $\mathbf{M}_{mn}^{1}$ and $\mathbf{N}_{mn}^{1}$ denote the VSWFs of the first kind, while $\mathbf{M}_{mn}^{3}$ and $\mathbf{N}_{mn}^{3}$ represent those of the third kind [1]. The expansion coefficients for the incident field, (*a_mn_*, *b_mn_*), are determined from Equation 1 as:

 (S3)

where *d_n_* = [(2*n*+1)/(4π*n*(*n*+1))]^1/2^ is a constant, (*L_ρ_*, *L_φ_*) are the radial and azimuthal components of input field **L**, $\pi_{mn}$ and $\tau_{mn}$ are functions involving the associated Legendre polynomials, and **r**_0_ denotes the position coordinate of the particle. Subsequently, the expansion coefficients of the scattered field, (*p_mn_*, *q_mn_*), are readily obtained via the T-matrix transformation [1]:

$\left[\begin{matrix} p_{mn} \\ q_{mn} \end{matrix}\right]=\left[\begin{matrix} T_{mnm'n'}^{11} & T_{mnm'n'}^{12} \\ T_{mnm'n'}^{21} & T_{mnm'n'}^{22} \end{matrix}\right]\left[\begin{matrix} a_{m'n'} \\ b_{m'n'} \end{matrix}\right] .$ (S4)

We consider an isotropic chiral medium characterized by its relative permittivity *ε*_2_, relative permeability *μ*_2_, and a dimensionless chirality parameter *κ* that quantifies its handedness. Reversing the sign of *κ* is equivalent to taking the material’s mirror image. This medium supports two eigenwaves—left- and right-handed circularly polarized—with effective refractive indices given by $n_{\pm}=\sqrt{\varepsilon_{2}\mu_{2}}\pm\kappa$. Due to broken parity symmetry, the T-matrix of a chiral sphere is non-diagonal in the parity basis, while still respecting the symmetry imposed by electromagnetic reciprocity, with elements [2, 3]:

$\begin{matrix} T_{mnm'n'}^{11}=-\delta_{mm'}\delta_{nn'}b_{n} , \\ T_{mnm'n'}^{22}=-\delta_{mm'}\delta_{nn'}a_{n} , \\ T_{mnm'n'}^{12}=-\delta_{mm'}\delta_{nn'}c_{n} , \\ T_{mnm'n'}^{21}=T_{mnm'n'}^{12} . \end{matrix}$ (S5)

The matrix elements incorporate the generalized Mie coefficients *a_n_*, *b_n_*, and *c_n_* for chiral media, as defined by Bohren and Huffman [4]:

$\begin{matrix} a_{n}=\frac{V_{n}(R)A_{n}(L)+V_{n}(L)A_{n}(R)}{W_{n}(L)V_{n}(R)+W_{n}(R)V_{n}(L)} , \\ b_{n}=\frac{W_{n}(L)B_{n}(R)+W_{n}(R)B_{n}(L)}{W_{n}(L)V_{n}(R)+W_{n}(R)V_{n}(L)} , \\ c_{n}=\frac{W_{n}(R)A_{n}(L)-W_{n}(L)A_{n}(R)}{W_{n}(L)V_{n}(R)+W_{n}(R)V_{n}(L)}, \end{matrix}$ (S6)

with

$\begin{matrix} W_{n}(J)=N\psi_{n}(N_{J}x)\xi'_{n}(x)-\xi_{n}(x)\psi'_{n}(N_{J}x) , \\ V_{n}(J)=\psi_{n}(N_{J}x)\xi'_{n}(x)-N\xi_{n}(x)\psi'_{n}(N_{J}x) , \\ A_{n}(J)=N\psi_{n}(N_{J}x)\psi'_{n}(x)-\psi_{n}(x)\psi'_{n}(N_{J}x) , \\ B_{n}(J)=\psi_{n}(N_{J}x)\psi'_{n}(x)-N\psi_{n}(x)\psi'_{n}(N_{J}x) . \end{matrix}$ (S7)

Here, *J* denotes either *L* or *R*, and *x* = *k*_1_*a*. The functions *ψ**_n_* and *ξ_n_* represent the Riccati-Bessel functions (related to the spherical Bessel and Hankel functions of the first kind, respectively). The relative refractive indices *N_L_*, *N_R_*, and the mean refractive index *N* are defined as$N_{L}=(\sqrt{\varepsilon_{2}\mu_{2}}+\kappa)/\sqrt{\varepsilon_{1}\mu_{1}}$,$N_{R}=(\sqrt{\varepsilon_{2}\mu_{2}}-\kappa)/\sqrt{\varepsilon_{1}\mu_{1}}$, and $N=(N_{L}+N_{R})/2$. Consequently, the optical force can be expressed in terms of these field coefficients as:

$\begin{matrix} <F_{x}+iF_{y}>=-\frac{\varepsilon_{1}}{4k_{1}^{2}}\sum_{n=1}^{\infty} \sum_{m=-n}^{n} [c_{1}(a_{m,n}q_{m+1,n}^{*}+b_{m,n}p_{m+1,n}^{*}+p_{m,n}b_{m+1,n}^{*}+q_{m,n}a_{m+1,n}^{*}+2p_{m,n}q_{m+1,n}^{*}+2q_{m,n}p_{m+1,n}^{*}) \\ -ic_{2}(a_{m,n}p_{m+1,n+1}^{*}+p_{m,n}a_{m+1,n+1}^{*}+b_{m,n}q_{m+1,n+1}^{*}+q_{m,n}b_{m+1,n+1}^{*}+2p_{m,n}p_{m+1,n+1}^{*}+2q_{m,n}q_{m+1,n+1}^{*}) \\ -ic_{3}(a_{m-1,n+1}p_{m,n}^{*}+p_{m-1,n+1}a_{m,n}^{*}+b_{m-1,n+1}q_{m,n}^{*}+q_{m-1,n+1}b_{m,n}^{*}+2p_{m-1,n+1}p_{m,n}^{*}+2q_{m-1,n+1}q_{m,n}^{*})] , \\ <F_{z}>=-\frac{\varepsilon_{1}}{2k_{1}^{2}}\sum_{n=1}^{\infty} \sum_{m=-n}^{n} \mathrm{Im}[c_{4}(a_{m,n+1}p_{m,n}^{*}+p_{m,n+1}a_{m,n}^{*}+b_{m,n+1}q_{m,n}^{*}+q_{m,n+1}b_{m,n}^{*}+2p_{m,n+1}p_{m,n}^{*}+2q_{m,n+1}q_{m,n}^{*}) \\ +ic_{5}(q_{m,n}a_{m,n}^{*}+b_{m,n}p_{m,n}^{*}+2q_{m,n}p_{m,n}^{*})] , \end{matrix}$ (S8)

where

$\begin{matrix} c_{1}=\frac{\sqrt{(n-m)(n+m+1)}}{n(n+1)}, c_{2}=\sqrt{\frac{n(n+2)(n+m+1)(n+m+2)}{(n+1)^{2}(2n+1)(2n+3)}}, c_{3}=\sqrt{\frac{n(n+2)(n-m+1)(n-m+2)}{(n+1)^{2}(2n+1)(2n+3)}}, \\ c_{4}=\sqrt{\frac{n(n+2)(n-m+1)(n+m+1)}{(n+1)^{2}(2n+1)(2n+3)}}, c_{5}=\frac{m}{n(n+1)} . \end{matrix}$ (S9)

Figure S2 displays the optical force distributions for the S and R enantiomers (*a* = 0.25 μm, *κ* = ±0.5) in the *xz*-plane at different positions (*x*_1_, *x*_2_) within the dual optical needle field. When the optical needle is located at (−0.5, 0.5) μm, the symmetry of the optical forces acting on the enantiomers is somewhat diminished, particularly near the optical axis. As the separation distance increases, the symmetry of the optical forces improves. In all three cases, the enantiomers remain transversely trapped by their respective optical needles and experience an optical pulling force. Although the optical force on the enantiomers slightly decreases with increasing distance, this does not compromise the enantioselective transport, as later analysis confirms. The adjustment of the distance between the two optical needles thereby provides flexible control over the separation process.

**Figure S2.** Optical force distributions in the *xz*-plane for the S and R enantiomers on different positions (*x*_1_, *x*_2_) of the dual optical needles.

**Supplementary Section 3: Move trajectories of chiral particles**

The dynamics of a Brownian particle with mass *m* in a fluid (viscous drag coefficient *γ*) under an optical force field **F(r)** are governed by the Langevin equation [5, 6]:

$m\frac{d^{2}r}{dt^{2}}=F(r)-\gamma\frac{dr}{dt}+\sqrt{2\gamma k_{B}T}\xi(t) ,$ (S10)

where ***ξ***(*t*) is a zero-mean, delta-correlated Gaussian white noise vector satisfying <*ξ_α_*(*t*)*ξ_β_*(*t*′)> = *δ_αβ_δ*(*t−t*′). We choose a simulation time step Δ*t* that is much larger than the particle’s momentum relaxation time, *τ* = *m*/*γ*. Under this condition, the inertial term is negligible, and Equation S10 reduces to the overdamped Langevin equation [7]:

$\frac{dr}{dt}=\frac{1}{\gamma}F(r)+\sqrt{2D}\xi(t) ,$ (S11)

where *D* = *k_B_*T/*γ* is the diffusion coefficient. Equation S11 is integrated numerically using the Euler-Maruyama scheme [8]. The particle trajectory is updated iteratively as:

$r_{i+1}=r_{i}+\frac{\Delta t}{\gamma}F(r_{i})+\sqrt{2D\Delta t}W_{i} ,$ (S12)

where **r***_i_* ≡ **r**(*t_i_*), *t_i_* = *i*Δ*t*, and **W***_i_* is a vector of independent standard normal random variables 𝒩(0,1). Thus, starting from an initial position (*z*_0_, *x*_0_) and given the optical force field (*F_z_*, *F_x_*) and the relevant thermodynamic parameters, the chiral separation and optical pulling dynamics are efficiently simulated.

The motion of chiral particles is governed by multiple factors. To elucidate these influences, Figure S3 presents the simulated trajectories of enantiomer pairs under various conditions. In Figures S3a and S3b, the input power and particle properties are held constant (*P* = 100 mW, *a* = 0.25 μm, *κ* = ±0.5) to analyze the effects of the transverse separation and longitudinal transport distances. In Figure S3a, with the dual optical needles positioned at (*x*_1_, *x*_2_) = (−3, 3) μm, the S and R enantiomers are initially trapped on their respective handedness-matched optical needles at *x* = −3 μm and *x* = 3 μm. They are subsequently transported in the negative *z*-direction to *z* = −4 μm over a simulation duration of 0.5 s. When the initial position is changed to (26, 0) μm, as in Figure S3b, the S and R enantiomers are successfully transported to final positions of (−28, −1) μm and (−28, 1) μm, respectively, after 0.9 s. These results confirm that both the enantiomer separation distance and the axial transport distance are highly adjustable. In Figures S3c and S3d, the optical needle positions remain fixed, but the input power is increased to 200 mW with a simulation time of 0.35 s. This configuration examines scenarios with either a lower chirality parameter (*κ* = ±0.05, *a* = 0.25 μm) or a smaller particle size (*a* = 0.15 μm, *κ* = ±0.5). In both cases, the enantiomers are effectively separated onto their respective optical needles and undergo negative transport over the predetermined distance. Therefore, appropriately increasing the input power facilitates effective enantioselective transport for particles with relatively low chirality or small size.

**Figure S3.** Simulated trajectories of S (tangerine) and R (blue) enantiomers in the *xz*-plane under four distinct conditions: (a) Dual optical needles positioned at (*x*_1_, *x*_2_) = (−3, 3) μm. (b) Long-range transport from *z* = 26 μm to *z* = −28 μm. (c) A lower chirality parameter of *κ* = ± 0.05. (d) A smaller particle radius of *a* = 0.15 μm.

**References:**

1. M. I. Mishchenko, L. D. Travis, and A. A. Lacis, *Scattering, absorption, and emission of light by small particles* (Cambridge university press, 2002).
2. F. Patti, R. Saija, P. Denti, et al., “Chiral optical tweezers for optically active particles in the T-matrix formalism,” *Scientific Reports* 9, no. 1 (2019): 29.
3. M. Li, S. Yan, Y. Zhang, X. Chen, and B. Yao, “Optical separation and discrimination of chiral particles by vector beams with orbital angular momentum,” *Nanoscale Advances* 3, no. 24 (2021): 6897-6902.
4. C. F. Bohren and D. R. Huffman, *Absorption and scattering of light by small particles* (John Wiley & Sons, 2008).
5. D. L. Ermak and J. A. McCammon, “Brownian dynamics with hydrodynamic interactions,” *The Journal of Chemical Physics* 69, no. 4 (1978): 1352-1360.
6. G. Volpe and G. Volpe, “Simulation of a Brownian particle in an optical trap,” *American Journal of Physics* 81, no. 3 (2013): 224-230.
7. T. Horai, H. Eguchi, T. Iida, and H. Ishihara, “Formulation of resonant optical force based on the microscopic structure of chiral molecules,” *Optics Express* 29, no. 23 (2021): 38824-38840.
8. S. Kieninger and B. G. Keller, “Path probability ratios for Langevin dynamics—Exact and approximate,” *The Journal of Chemical Physics* 154, no. 9 (2021): 094102.
